# A Strategy to Assess Quality Consistency of Drug Products

**DOI:** 10.3389/fchem.2019.00171

**Published:** 2019-03-26

**Authors:** Shu Y. Qi, Shang C. Yao, Li H. Yin, Chang Q. Hu

**Affiliations:** ^1^Division of Antibiotics, Institute for Chemical Drug Control, National Institutes for Food and Drug Control, Beijing, China; ^2^State Key Laboratory of Biochemical Engineering, Institute of Process Engineering, Chinese Academy of Sciences, Beijing, China

**Keywords:** quality consistency, critical quality attribute, quality standard, process variation, assessment strategy, drug product

## Abstract

Herein, we aimed to develop a strategy to assess quality consistency of a drug product, with a focus on two typical cases of injection. Multi-variable analysis using a sequencing combination of factor analysis, one-way analysis of variance and cluster analysis identified all potential Critical Quality Attributes (CQAs) for each manufacturing process, which were identified from the attributes of quality standard (QAs) using supervised (cefazolin sodium pentahydrate, α-CEZ-Na) or unsupervised (cephathiamidine, CETD) analysis. All CQAs from QAs were applied to set up an integrated index, quality consistency attribute (QCA), to evaluate product quality consistency in a specific aspect. Meanwhile, real-time analysis by chemometrics-assisted near-infrared spectroscopy (NIR) was used to obtain useful information corresponding to the CQAs from the process attributes (PAs) of some of the critical processes. The quantitative results of characteristic signals of NIR by multiple linear regression was defined as the process consistency attribute (PrCA), and was used to assess the product quality consistency in another aspect. Therefore, either values of QCA or PrCA displayed sensitivity to changes in product quality, allowing us to establish a strategy with strong practicality, comprehensiveness and visualization to demonstrate the quality consistency of a specific product. Such strategy is not only conducive to the improvement of quality standards, but to the retrospective investigation of manufacturing processes which ultimately allowed maintenance of product consistency.

## Introduction

The quality of a drug (medicinal) product should be strictly controlled to ensure its safety and efficacy; this serves as a basis for formulating quality standards. Generally, the quality standards of drugs contain attributes such as drug definition, identification, assay, and impurities. Many other attributes such as crystal form, particle size, and bacterial contamination are significantly influenced by changes in the manufacturing process. These changes may in turn affect the quality of the drugs as discussed in the 60th Technical Report of the Parenteral Drug Association (Parenteral Drug Association. TR60; 2017); however, every attribute does not play a prominent role when assessing product quality. The ICH Q8 (R2) defines the Critical Quality Attribute (CQA) as the physical, chemical, biological, or microbiological property or characteristic that should be within an appropriate limit, range, or distribution to ensure the desired product quality is achieved (International Conference on Harmonisation, 2009). The ICH Q9 indicates that the investigation of CQAs is vital to quality risk assessment (International Conference on Harmonisation, 2005); therefore, it is important to delineate potential CQAs from all attributes. This process is however difficult, time-consuming, and costly to perform by an interdisciplinary team, which must include experts from the appropriate fields (e.g., quality unit, business development, engineering, regulatory affairs, production operations, sales, and marketing, legal, statistics and clinical). A quality assessment was performed with all attributes constituting the quality product profile separated into two parts: attributes included in the quality standards, defined as Quality Attributes (QAs); and other attributes influenced by process consistency, defined as Process Attributes (PAs).

For QAs of a product, a testing method and qualification limit have been derived for each attribute. Once an attribute exceeds its limit, this indicates that the corresponding product has a certain degree of quality defects and these samples were not considered in the present research. To quantify the influence of QAs on drug quality, multi-variable analysis methods are used to identify the sensitivity of a certain attribute to the quality variation of a product. The higher the recognition rate of an attribute to specific changes in product quality, the more significant this attribute is in quality consistency. For each process, only the most significant attribute is regarded as the CQA to display its process change. The quality consistency of a product would then be evaluated by integrating the contributions of the CQAs. Based on the performance of CQAs, the relationship between quality variation and process change could then be established.

Different drug (medicinal) products have different PAs, which is dependent on the manufacturing procedure, technical conditions and parameters. After accumulating CQAs from QAs, the corresponding processes based on an in-depth understanding of the production process may be derived. In addition, the strategy for deriving CQAs from PAs was stated in detail in our previous study (Qi et al., 2018). Together, the CQAs of QAs and PAs, and the relationship between CQAs and critical process parameters (CPPs) could be established to assist in the later development of a quality control strategy.

Two drugs were selected as typical cases to develop and test the above technique: cefazolin sodium pentahydrate (α-CEZ-Na) from Shenzhen Gosun Pharmaceutical Co., Ltd., whose production conditions change in different processing periods; and cephathiamidine (CETD) from Guangzhou Baiyunshan Pharmaceutical Co., Ltd., whose production conditions are kept the same. These two products were powder for injections obtained by direct packing of raw materials which were produced by an especial crystallization of active ingredient. Thus their product quality depended mainly on the consistency of crystallization process. Here, the PAs from crystallization process would come into greater focus.

## Materials and Methods

### Samples

The 150 samples of α-CEZ-Na from three processing periods (Period 1: July 2014 – January 2015 and September 2015; Period 2: November 2016—December 2016; Period 3: January 2017—March 2017) were provided by Shenzhen Gosun Pharmaceutical Co., Ltd. (Guangdong, China; Gosun). To amplify the differences between products, the former 50 samples from Period 1 (Set 1) were produced in the old manufacturing site, and the middle 50 samples from Period 2 (Set 2) and the remaining 50 samples from Period 3 (Set 3) produced in the new manufacturing site. The quality of every α-CEZ-Na sample complied with the Japanese Pharmacopeia, including 28 QAs (e.g., specific rotation, absorption coefficient, pH, water, related substances (Impurity A, B, C, F, G, H, I, J, K, L, M, and N, maximum unspecified, and total), polymer, residual solvents (dimethylacetamide, acetone, isopropanol, and dichloromethane), sub-visible particles (≥10 um and ≥25 um), density (bulk and tapped), and assay).

The 96 samples of CETD from different batches were provided by Guangzhou Baiyunshan Pharmaceutical Co., Ltd. (Guangzhou, China). The quality of every CETD sample complied with the Chinese Pharmacopeia, including 23 QAs (e.g., specific rotation, pH, water, related substances (Impurity A, B, C, and D, maximum unspecified, and total), residual solvents (methanol, ethanol, acetone, and dichloromethane), bacterial endotoxins, sub-visible particles (≥10 um and ≥25 um), and assay).

### NIR

Samples were directly scanned in vials using a Fourier transform NIR integrating sphere (MPA, Bruker, Switzerland). The scan wavelength range and resolution were set to 12000–4000 cm^−1^ and 8 cm^−1^, respectively. All spectra were obtained by averaging the results of 32 scans, and 6 spectra of the same sample averaged to obtain a representative mean spectrum.

### Data Handling

For the drug products from the same variety and dosage form, their production conditions were focused on the manufacturer, manufacturing site, facility and equipment, and their operating parameters. The details of major data handling methods used to assess quality consistency are given below.

I. Z-scores

For some multivariate techniques such as multidimensional scaling and cluster analysis, the concept of distance between the units in the data is often of considerable interest and importance. When the variables in the multivariate data set are on different scales, it is more feasible to calculate the distances after some form of standardization (Everitt and Hothorn, 2011). Z-scores is a frequently used method to normalize data; its standard score of a raw *x* is calculated as

(1) $$\begin{array}{l} \text{z}=\left(x-\mu \right)/\sigma \end{array}$$

where μ is the mean of the population, and σ is the standard deviation of the population. Here, all values of QAs were transformed by Z-scores.

II. Factor Analysis

Factor analysis is a statistical method used to describe variability among observed, correlated variables for a potentially lower number of unobserved variables called factors. It is one of the most commonly used inter-dependence techniques and is used when the relevant set of variables shows a systematic inter-dependence and the objective is to find the latent factors that create a commonality. To distinguish from principal component analysis (PCA), factor analysis is clearly designed with the objective to identify specific unobservable factors from the observed variables (Jolliffe, 2002; Bartholomew et al., 2008). The feasibility of this method was tested using Kaiser-Meyer-Olkin measure of sampling adequacy (KMO>0.7) and Bartlett's test of sphericity (*P* < 0.05), and the extraction and rotation methods were PCA and Varimax with Kaiser Normalization, respectively. To reduce data dimensions, all QAs standardized by Z-scores were used in factor analysis to determine the least number of factors which could account for the common variance (correlation) of all variables.

III. Cluster Analysis

Cluster analysis is the task of grouping a set of objects where objects in the same group (called a cluster) are more similar (in some sense) to each other than to those in other groups (clusters). In this study, hierarchical cluster analysis (HCA) (Hedegaard et al., 2011) using Ward's method was usually applied to classify the samples based on Squared Euclidean. Its classification results for the target variables were used in the unsupervised data analysis when groups of samples were unknown. If the groups were given according to changes in production conditions, the importance of the target variables was determined by matching degree between clustering results and real groups; the above matching rate was defined as the recognition rate of changes (RRC) to show the level of importance of some attributes to product variation.

IV. Discriminant Analysis

Discriminant analysis (DA) is used when groups are known priori (unlike in cluster analysis). Each case must have a score for one or more quantitative predictor measures, and a score for group measure (Bökeoglu Cokluk and Büyüköztürk, 2008). Multiple linear regression (MLR), a quantitative method, was employed for discriminant analysis (MLR-DA), which was favorable for the visual display of results.

V. One-way analysis of variance (One-way ANOVA)

In statistics, one-way ANOVA is a technique used to compare the mean of two or more samples (using the F distribution) (Montgomery, 2001; Howell, 2002). Here, a computer typically determined the *p*-value (*P* < 0.05) from F which determines whether the attributes produced significantly different results when responding to the changes in production conditions. If the result was significant, then the corresponding attributes provisionally had validity. In addition, Fisher's Least Significant Difference test (LSD) was used for multiple comparisons of the above attributes. It is well-known that the products under the same production condition have higher quality consistency than those under different production conditions. If the LSD result of a specific attribute conformed to this phenomenon, this attribute was listed as a possible CQA.

Depending on the studied processes, all possible CQAs were separated into several parts by different manufacturing processes. Under the same process, each possible CQA was applied to classify all samples by HCA (Ward's method), and their RRCs were determined and compared to find the highest. For a production process, the attribute with the highest recognition rate of changes (RRC_max_) was defined as the CQA in this process.

VI. Quality Consistency Attribute (QCA)

The entire operation of drug production is composed of several manufacturing processes. All QAs, a series of attributes given by the quality standard, should be separated into several parts based on different manufacturing processes. For each manufacturing process, finding at least one CQA to characterize its production status is crucial. It is thus reasonable to synthesize all CQAs and their RRCs to form an assessment index for product quality consistency. Here, this index was defined as the quality consistency attribute (QCA) and was calculated using the following formula:

(2) $$\begin{array}{l} \text{QCA=}\overset{N}{\underset{i=1}{\sum }}W_{i}|CQA_{i}|\text{=}\overset{N}{\underset{i=1}{\sum }}\frac{RRC_{i}}{\overset{N}{\underset{i=1}{\sum }}RRC_{i}}|CQA_{i}| \end{array}$$

where *W*_*i*_ was the contribution weight of *CQA*_*i*_ to characterize product quality variation.

## Results and Discussion

### α-CEZ-Na

#### Assessment Based on QAs

It should be noted that the production conditions were changed for different sets of α-CEZ-Na; the samples from the old manufacturing site (Set 1) were defined as the group of Y_1_ = 0, and samples from the new manufacturing site (Set 2 and Set 3) defined as the group of Y_2_ = 1. The assessment of the QAs of α-CEZ-Na was performed under supervised analysis.

All 28 QAs of α-CEZ-Na normalized by Z-scores were analyzed by one-way ANOVA, and for 10 of these QAs, specific rotation, water, related substances (Impurity H, I, M, and N, and total), acetone, sub-visible particles (≥25 um), and tapped density were identified as possible CQAs that were significantly affected by sample group changes.

Depending on the process under scrutiny, these 10 attributes were separated into three parts: (1) crystallization process, (2) drying process, and (3) plant cleanliness for Good Manufacturing Practice (GMP). (1) During the process of crystallization, the optimum temperature range and duration for salt formation, crystallization, and recrystallization affect the content of various process impurities (Impurity H, I, M, and N); therefore influencing the specific rotation and total impurity. Meanwhile, the addition rate of isopropanol and temperature could also affect the particle size of crystals; thus affecting the tapped density of the product. Among these 7 attributes, specific rotation had an RRC_max_ of 94%. (2) In the process of drying, acetone was used to wash and remove isopropanol from the crystal product. Acetone and excess water were then removed by controlling the drying temperature and pressure. If these two parameters were not properly controlled, variation of acetone and water content between different batches of products would result. Among these 2 attributes, water had an RRC_max_ of 80%. (3) The differences in the cleanliness requirement for GMP would affect the amount of additional dust particles (sub-visible particles (≥25 um)) in the final product. According to the low RRC of sub-visible particles (≥25 um) (<60%), it was indicated that the variation of this attribute was not significant in influencing the quality of products from varied manufacturing sites. Thus, specific rotation and water were identified as the two CQAs for characterizing the crystallization process and drying process, respectively; therefore, these features can be used to calculate the QCA of α-CEZ-Na using Equation (2) in efforts to assess its quality.

**Figure 1:**
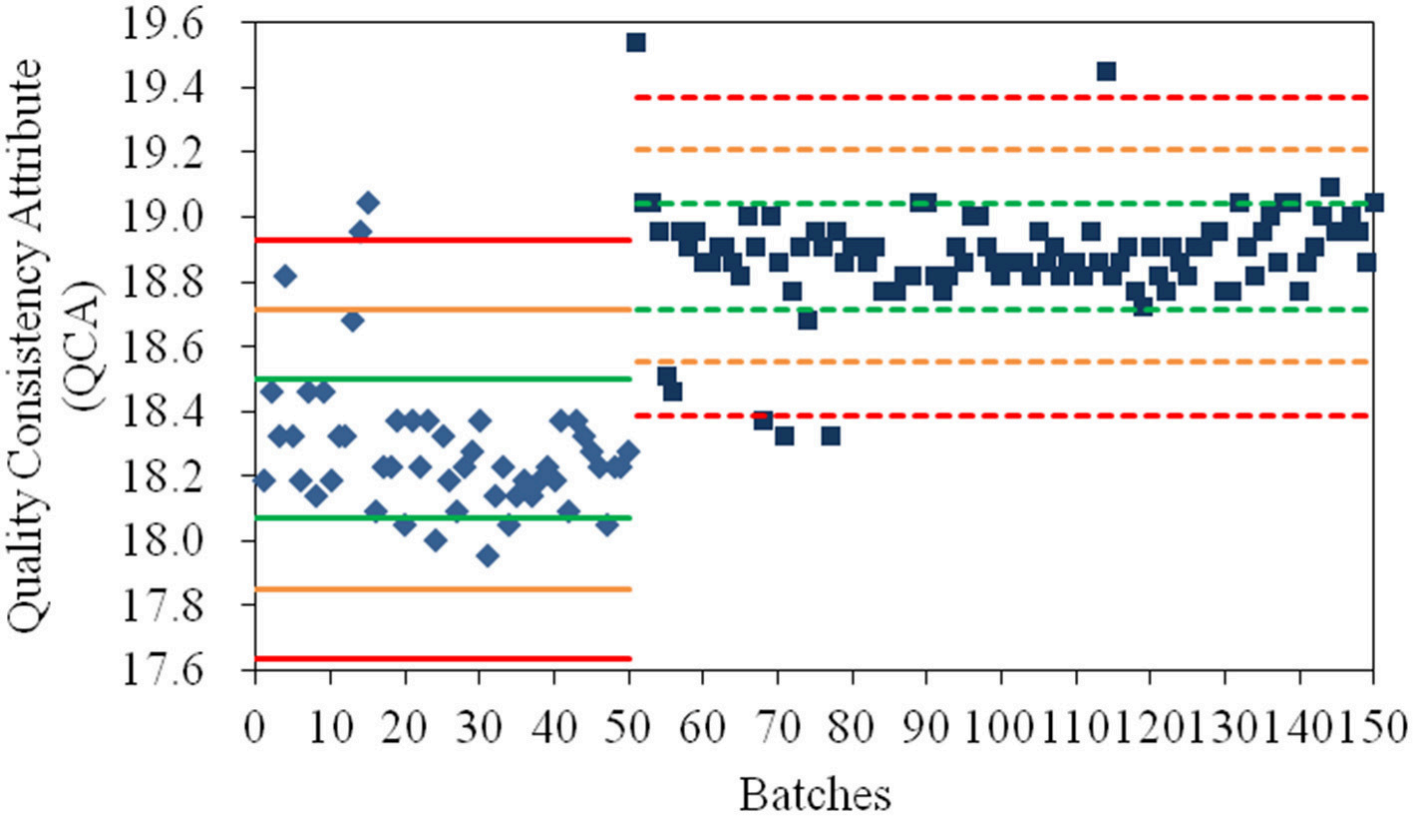
The values for the quality consistency attribute (QCA) for all α-CEZ-Na samples. Batches 1–50 marked by ♦ were the samples from the old manufacturing site, and Batches 51–150 marked by ■ were samples from the new manufacturing site. The green, yellow and red mark lines were, respectively the μ ± δ, μ ± 2δ, and μ ± 3δ of the QCA values of Batches 1–50 (solid-line) and Batches 51–150 (dashed-line).

As shown in Figure 1, the values for QCA could directly determine whether a quality risk existed in a product. In addition, its severity of risk could be detected by the Principle μ ± Nδ (*N* = 1, 2, 3), where μ and δ were the mean and standard deviation of the QCA values of samples from the same manufacturing site. The products whose QCA values were within the range of μ ± δ (green line) had high quality consistency. If the QCA value of a product exceeded the specification limit of μ ± 2δ (yellow line), especially the control limit of μ ± 3δ (red line), a risk of quality inconsistency was suggested and its production processes must be investigated. Based on the control limit of μ ± 3δ, 4, and 5% of products from the old manufacturing site and new manufacturing site, respectively, had high quality risk. When their values for the CQAs were investigated, the poor quality products from the old manufacturing site had higher values for specific rotation and water than normal; therefore, the crystallization process and drying process should be examined. In addition, the poor quality products from the new manufacturing site had both higher and lower specific rotation compared to normal; thus, the crystallization process was the only critical process that required further examination. The manufacturing process at the new manufacturing site was more stable than that at the old manufacturing site based on δ_2_ = 0.164 < δ_1_ = 0.215. The values for the QCA for α-CEZ-Na were useful to find the inconsistent products and the causes of quality variances, and to remove the deficiencies in the production process.

### Assessment Based on QAs and PAs

According to the RRCs for the CQAs in α-CEZ-Na QAs, the processes of crystallization and drying, especially the crystallization process, were the critical processes that required analysis to elucidate the resulting quality variance. Our previous study (Qi et al., 2018) indicated that variations in polymorph proportions could be defined as CQAs in PAs to reveal the consistency in the pharmaceutically important crystallization process; these could also be quantified using near infrared spectroscopy (NIR) with the assistance of chemometrics. As the polymorph proportions had characteristic responses around 4339, 4431, and 5280 cm^−1^ in the NIR spectra, their spectral signals were applied as variables and the groups (Y_1_ = 0 and Y_2_ = 1) used as the dependent variable in the MLR-DA. The predicted values of the above model were defined as process consistency attribute (PrCA), another index in assessing quality consistency. It should be noted that the control limits for PrCA were 0 ± 0.5 and 1 ± 0.5 for samples from the old and new manufacturing sites, respectively.

**Figure 2:**
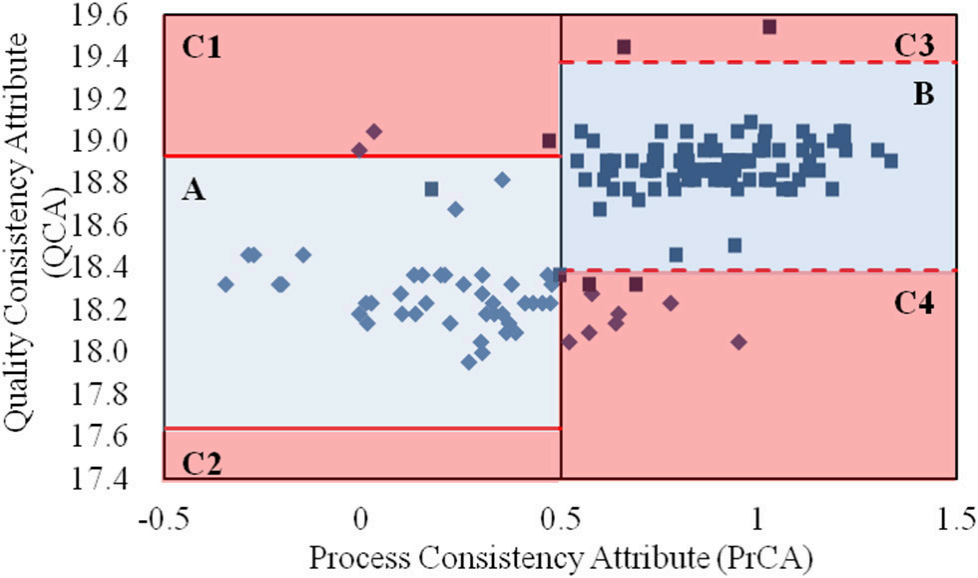
The values for the quality consistency attribute (QCA) and process consistency attribute (PrCA) for all α-CEZ-Na samples. The 50 samples marked by ♦ were from the old manufacturing site, and the 100 samples marked by ■ were from the new manufacturing site. Their QCA values were, respectively used to calculate the control limit μ ± 3δ, which were marked by red solid-line and red dashed-line. And the boundaries of Area A, B, and C1-4 were defined by the control limit μ ± 3δ of QCA as well as the control limit 0 ± 0.5 and 1 ± 0.5 of PrCA.

By integrating the values for the QCA and PrCA of α-CEZ-Na in Figure 2, the quality consistency of products could be analyzed in its entirety. According to the control limits for PrCA, 16% of samples from the old manufacturing site had high quality risk; however, the proportion reduced to 0.7% at the new manufacturing site. Based on the values of QCA and PrCA, the products located in Area A (82% from the old site) and Area B (95% from the new site) had good quality consistency within their control limits. The great improvement at the manufacturing level of the new site was evident. However, unusual changes existed in those located in Area C, 1–4. For samples from the old manufacturing site in Area C1, their CQAs in QAs (specific rotation and water) were abnormal, indicating the unusual degradation of drug molecules or unfavorable of drying. Samples from the new manufacturing site in Area C1 however had their proportions of folded and stretched conformations of API significantly changed, indicating the unexpected variation of solvent ratio or temperature. For samples in Area C3 or C4, the phenomena were contrary to that observed in Area C1. Thus, a comprehensive assessment strategy with both QAs and PAs could intuitively depict the quality variation of a target product, as well as directly demonstrate the causes of quality variation.

Interestingly, the similarity in the assessment results between QAs and PAs was 86.7%, indicating that the assessment by QAs could largely replace the comprehensive assessment strategy for α-CEZ-Na, and control of the crystallization process was key to ensuring quality consistency of the α-CEZ-Na products.

### CETD

#### Assessment Based on QAs

**Figure 3:**
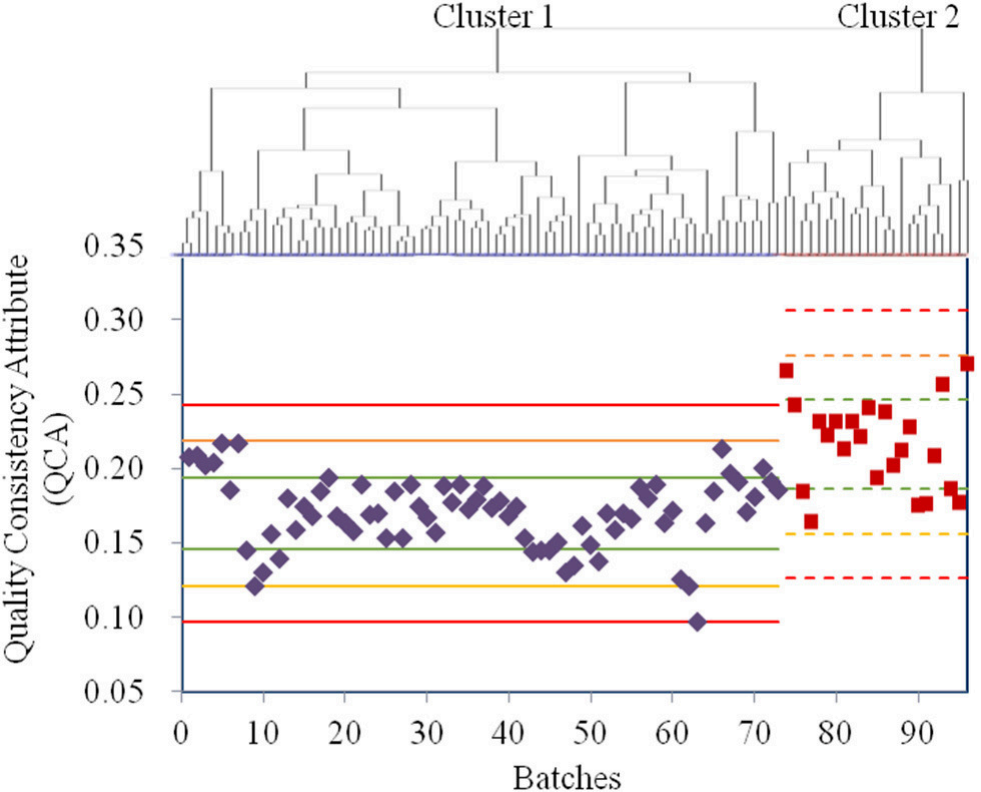
The results for the quality assessment for all CETD samples based on the quality attributes from its quality standard. The cluster analysis was conducted by HCA with Ward's method, and all the samples were classified into Cluster 1 (73 purple ones) and Cluster 2 (23 red ones). The values of quality consistency attribute (QCA) of Cluster 1 and Cluster 2 were, respectively used to calculate the μ ± δ (green line), μ ± 2δ (yellow line), and μ ± 3δ (red line), and their mark lines were differed by solid-line and dashed-line.

Owing to the use of the same production conditions, the CETD samples were not assigned as the control variables in one-ANOVA, an unsupervised analysis. Thus, all 23 QAs of CETD were first processed using factor analysis to reduce the variable dimensions; the extracted variables were then used in a cluster analysis (Figure 3). According to Figure 3, the sample qualities from different batches were essentially the same; however, to determine potential CQAs, the samples were classified into two clusters. Following the procedure of a supervised analysis for α-CEZ-Na, total impurity was regarded as a CQA for the crystallization process with RRC of 74%. Ethanol was considered another CQA for the drying process with RRC of 82%. The QCA of CETD was calculated using **Equation (2)**. According to the Principle μ ± 3δ, samples from the two clusters were, for the most part, superimposable (Figure 3), indicating that the quality consistency of CETD was satisfactory based on its quality standard.

#### Assessment Based on QAs and PAs

**Figure 4:**
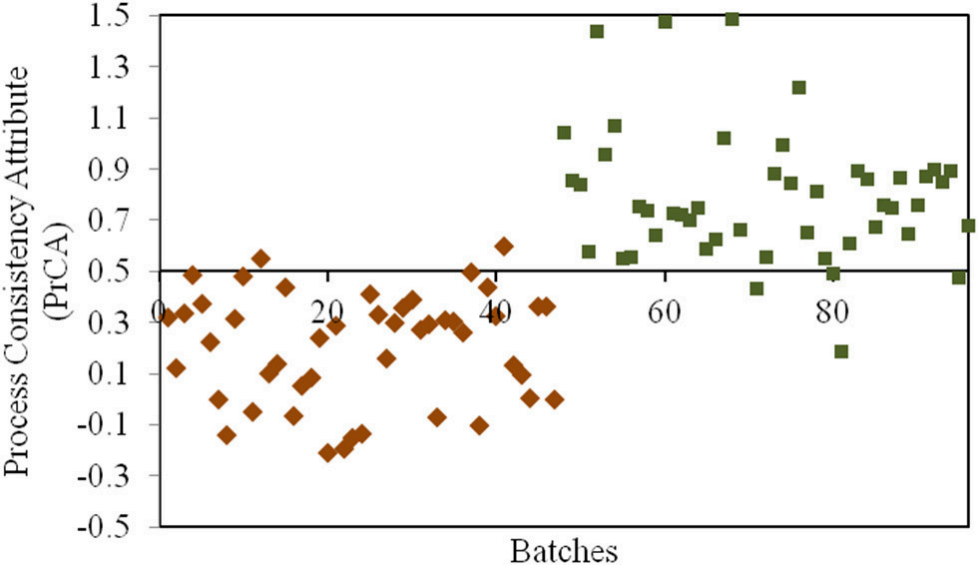
The results for the process consistency attribute (PrCA) of all CETD samples. Batches 1–47 marked by ♦ had a higher proportion of plate-like crystals to rod-like crystals (Group 1, Y_1_ = 0 ± 0.5); and Batches 48–96 marked by ■ had a lower proportion of plate-like crystals to rod-like crystals (Group 2, Y_2_ = 1 ± 0.5).

According to the assessment by QAs, crystallization process remained as the critical process for CETD. Our previous study (Qi et al., 2018) indicated that different proportions of plate-like and rod-like crystals could be defined as CQAs in PAs for CETD to reveal the consistency of the crystallization process. These could be quantified using near infrared spectroscopy (NIR) using the MLR-DA model at 5211, 5284, and 5369 cm^−1^ (Figure 4). For samples in Group 1 (Y_1_ = 0 ± 0.5), the proportion of plate-like crystals was higher than that of rod-like crystals; opposite results were obtained for samples in Group 2 (Y_2_ = 1 ± 0.5).

**Figure 5:**
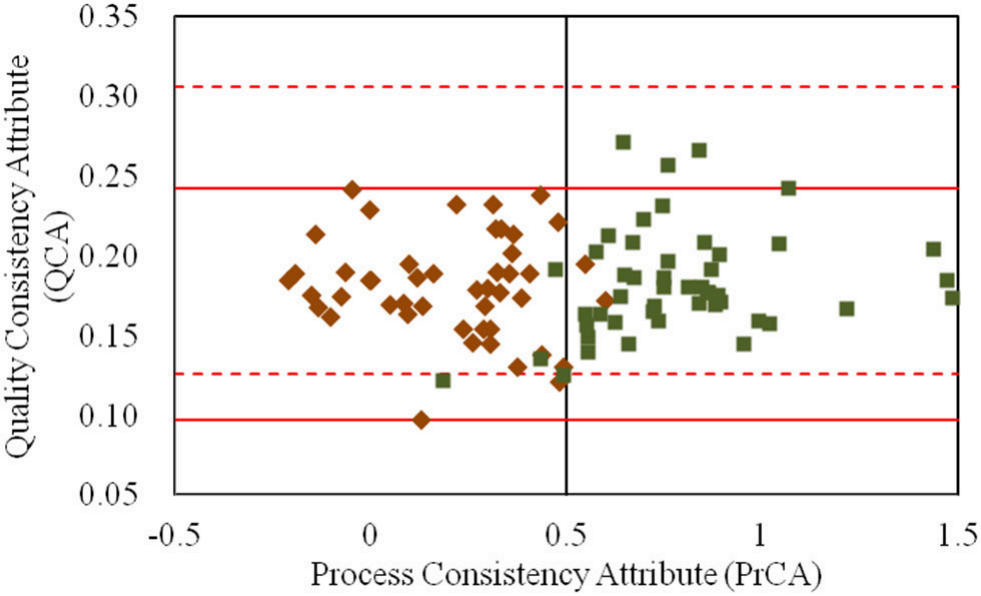
The values for the quality consistency attribute (QCA) and process consistency attribute (PrCA) for all CETD samples. The 47 samples marked by ♦ had a higher proportion of plate-like crystals to rod-like crystals (Group 1); and the 49 samples marked by ■ had a lower proportion of plate-like crystals to rod-like crystals (Group 2). Their QCA values were, respectively used to calculate the control limit μ ± 3δ, which were marked by red solid-line and red dashed-line.

As shown in Figure 5, the quality variations of products were mainly distinguished by PAs and not by QAs. It was indicated that the quality standard was insufficient to characterize the quality variation of the CETD product. Thus, to improve the quality standard of CETD, new quality attributes had to be extracted to characterize the changes of products with different crystal forms. As both the physical and chemical properties of products holding different crystal forms would be varied, an ideal crystallization process should only form a single crystal. It was therefore necessary to determine the best crystal form, and then control the trend of target crystallization by the control limits for the PrCA.

## Conclusions

**Chart 1:**
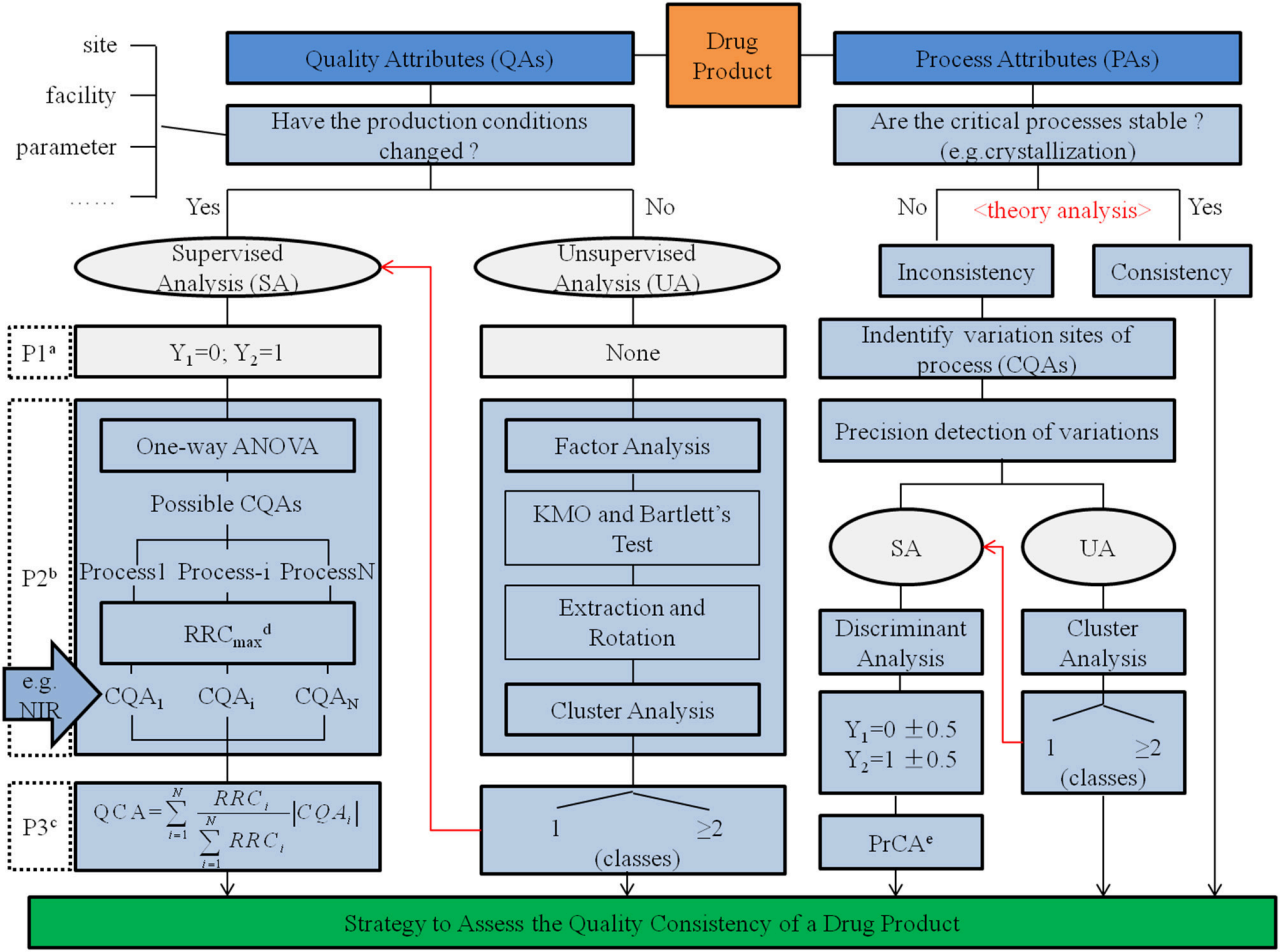
Strategy to assess quality consistency of a drug product. a The procedure to identify groups. Here, the samples from one type of production conditions was represented using Y_1_ = 0 and samples from another production condition type represented using Y_2_ = 1. b The procedure to reduce the data dimensions. c The procedure to assess quality consistency using the index of quality consistency attribute (QCA). d The recognition rate of changes (RRC) is the matching rate between cluster results given by cluster analysis and real groups identified by the production conditions. RRC_max_ was the highest RRC of possible CQAs in a production process. e The process consistency attribute (PrCA) is the discriminant value calculated by multiple linear regression, another index to assess quality consistency.

Quality consistency of drug products was determined based on the utilization of consistency in the manufacturing process. Applying CQAs both from QAs in quality standard and PAs affected by changes in the manufacturing process served as an adequate and useful strategy to assess the quality consistency of drug products (Chart 1). For α-CEZ-Na, consistent results assessed by CQAs of QAs were verified in 86.7% of samples based on the assessment of quantified CQAs of PAs; however, for CETD, the results from QAs had to be formed from PAs. To improve the accuracy and practicability of the assessment strategy in deriving the quality consistency of drug products, jointly using attributes from the quality standard and manufacturing process proved to be an effective method. Depending on the rigorous and logical screen of chemometrics methods, the scientific integration of QAs and PAs could be realized to develop this systemic approach.

Meanwhile, through this strategy as shown in Chart 1, it is possible to improve the quality standard of a specific drug, as well as achieve a timely and targeted process feedback survey; these were helpful in the control of product quality and to the production attributes required for real-time release.

## Data Availability

The datasets generated for this study are available on request to the corresponding author.

## Author Contributions

SQ: design of experiments, data acquisition, analysis of data. SY: assisted analysis of attributes in quality standard. LY: assisted analysis of process attributes. CH: design of experiments, analysis of data.

### Conflict of Interest Statement

The authors declare that the research was conducted in the absence of any commercial or financial relationships that could be construed as a potential conflict of interest.
